# Applicability of Field Aerobic Fitness Tests in Soccer: Which One to Choose?

**DOI:** 10.3390/jfmk6030069

**Published:** 2021-08-18

**Authors:** Daniel Bok, Carl Foster

**Affiliations:** 1 Faculty of Kinesiology, University of Zagreb, 10000 Zagreb, Croatia; 2 Department of Exercise and Sport Science, University of Wisconsin-La Crosse, La Crosse, WI 54601, USA; cfosteruwl@gmail.com

**Keywords:** 30-15 intermittent fitness test, Yo-Yo intermittent recovery test, 20-m shuttle run test, University of Montreal track test, Vam Eval test, maximal aerobic speed, exercise prescription, validity, reliability, sensitivity, usefulness

## Abstract

A desire to make fitness testing cheaper and easier to conduct in a team-sport setting has led to the development of numerous field aerobic fitness tests. This has contributed to a growing confusion among strength and conditioning coaches about which one to use. The main aim of this narrative review was to examine the reliability, validity, sensitivity and usefulness of the commonly used field aerobic fitness tests and to provide practical guidelines for their use in soccer. The University of Montreal track test (UMTT) and Vam Eval test seem the best options for estimation of maximal oxygen uptake (VO_2max_) while the highest signal-to-noise ratio of the 30-15 intermittent fitness test (30-15IFT) suggests its superior sensitivity to track changes in fitness. The UMTT and 30-15IFT are the best solutions for prescription of long and short high-intensity interval training sessions, respectively. All field tests mostly present with marginal usefulness, but the smallest worthwhile change for UMTT or Vam Eval test, Yo-YoIRT2 and 30-15IFT are smaller than their stage increment making the improvement of only one stage in the test performance already worthwhile. Strength and conditioning coaches are advised to choose the test based on their specific purpose of testing.

## 1. Introduction

Fitness testing can be considered as a basic professional activity for sport scientists and strength and conditioning coaches [1,2]. It can be conducted for a variety of reasons including assessment of physiological capacities [1,3], talent identification and selection [4], training and performance monitoring [5], evaluation of training program effectiveness [1,6] and training prescription [7]. Due to these multipurpose requirements, one fitness test can hardly be used as an ideal tool able to provide useful information for all aspects of fitness testing. This has led to the development of numerous field aerobic fitness tests generally measuring the same generic fitness quality (i.e., maximal aerobic power), but with better or limited applicability for the specific purpose of testing. Sport-specific fitness tests, for example, appear to show greater ecological validity, but, on the other hand, have limited convergent validity. This makes them appropriate for specific-fitness assessment, but rather poor in assessment of basic fitness capacities (e.g., the maximal oxygen uptake (VO_2max_)) [8]. Similarly, some tests present with limited practical validity and can, therefore, hardly be used to accurately prescribe exercise which is probably the most important part of strength and conditioning coaches’ job [3].

Although laboratory incremental exercise test is considered a “gold standard” for testing VO_2max_ [9], field aerobic fitness tests have emerged as time and resource-saving alternatives. During the last four decades, several field tests, including the University of Montreal track test (UMTT) [10], along with its modification the Vam Eval test [11], multistage 20-m shuttle run test (20mSRT) [12], Yo-yo intermittent recovery test level 1 and 2 (Yo-YoIRT1 and 2) [8] and 30-15 intermittent fitness test (30-15IFT) [13] have gained popularity and are widely used in practice for the assessment of aerobic fitness. These tests are different in nature as they include multistage continuous straight-line [10,11], shuttle [12] and intermittent shuttle [8,13] runs to exhaustion. Due to differences in execution, these field tests provide different end-test speeds which are specific to the nature of the effort made during the test. Specifically, introducing changes of direction every 20 m into straight-line running yields higher oxygen uptake (VO_2_), heart rate, blood lactate concentration and Rating of Perceived Exertion responses [14,15] which leads to exhaustion at significantly lower end-test speeds during shuttle tests in comparison to incremental straight-line tests [16]. Similarly, omitting inter-effort recoveries while performing the 30-15IFT results in reaching exhaustion at significantly lower end-test speed [17]. For similar levels of aerobic fitness exhaustion will be reached at the lowest running speed in 20mSRT while UMTT or Vam Eval test, Yo-YoIRT and 30-15IFT will have their end-test speeds higher by approximately 2 km/h interval each. This would, for example, make the end-test speed of 30-15IFT approximately 6 km/h greater than the one reached in 20mSRT. Since velocity associated with VO_2max_ (vVO_2max_) is the preferred method for prescribing exercise intensity for high-intensity interval training (HIIT) [18] this measure should be obtained through a field test which closely mimics the locomotor activity of a certain HIIT format in order to make it usable for prescription. However, not all mentioned field tests are specific to the HIIT formats, so their end-test speeds cannot easily be used for training prescription purposes.

Field tests are very popular as they are less time-consuming and cheaper than tests performed in the laboratory. However, the large number of available field tests has contributed to a growing confusion among coaches about which one to use. All mentioned field tests were nominally created for aerobic fitness assessment. This has led coaches to believe that the tests are basically the same and that the choice can be made solely on preference. However, each test had been developed with a specific purpose and, as such, should be used if and when it has the best metric characteristics for a certain aspect of testing. Therefore, the main aim of this paper is to review the available scientific literature for the purpose of reporting and discussing the reliability, validity, sensitivity and usefulness of the most commonly used field aerobic fitness tests. This will enable the formation of practical guidelines for their proper use in soccer (football).

This paper used a narrative review format. In order to retrieve relevant scientific papers, we searched the Web of Science and PubMed databases using standard search criteria. After accounting for the already retrieved publications, the keywords mentioned in the abstract were used to search for additional scientific papers. Reference lists of retrieved articles and recently published reviews were examined to find additional papers not identified by the keywords-based search. Only full-text articles published in English were included in the review. The searching process included articles retrieved until 1 March 2021.

## 2. Assessment of Maximal Oxygen Uptake

The general importance of aerobic fitness in soccer is well documented [19,20]. However, even though strength and conditioning coaches seek information about their player’s VO_2max_, recent studies show that matching running performance might not be affected by aerobic fitness capacities [21,22,23] as much as previously reported [24,25,26,27]. Namely, it appears that playing position and game tactics are more important factors in determining how much a player will run during a match than physical fitness [22]. This is further supported by the findings that improvements in aerobic fitness do not necessarily reflect in improvements in high-intensity match running performance in young soccer players [28]. However, aerobically fitter players experience reduced individual running demands during the game which is beneficial in terms of reducing the overall fatigue and injury risk [29], as well as the impairment of technical performance [30]. So, even though aerobic fitness might not be the primary limiting factor for match running performance in soccer players [21,22,23], since players cover 9 to 12 km in total distance, perform 150 to 350 high-intensity running activities, and execute 50 to 100 accelerations above 2.5 m/s^2^ with 300 changes of directions during the match [31], adjusted aerobic conditioning should definitely be implemented in the overall training program. Well-developed aerobic fitness will enable players to perform their technical and tactical requirements with less physiological load [22] and to quickly recover between high-intensity efforts [32] which have been shown to be typical activities for team sports and especially soccer [27,33].

Probably the most important reason for fitness testing is the assessment of physical capacities and abilities [1,3]. Despite recent scientific evidence related to its association with soccer match performance, when it comes to aerobic or cardiorespiratory fitness, many strength and conditioning coaches focus on VO_2max_. However, it appears that rather than VO_2max_ per se, match running performance is more related to vVO_2max_ [23] and peak incremental test speed [21]. Both variables represent an integrated measure of VO_2max_ and running economy, and can therefore be considered an athlete’s peak aerobic locomotor ability [7]. This is further supported by the fact that repeated sprint ability (RSA) [34] shows much larger association with peak incremental test speed than VO_2max_ in team sport players [35]. Therefore, assessing peak aerobic locomotor ability seems to be much more important than VO_2max_ as it provides a more ecologically valid measure of aerobic fitness and can also be used for prescribing exercise.

Apart from being less important than end-test speed for assessment of aerobic fitness in team sports, another reason disputing the assessment of VO_2max_ through test equations is their low prediction accuracy due to the fact that the calculation presumes a standard running economy. While VO_2max_ can be reached in all field tests when executed to exhaustion [12,36,37], the calculation of VO_2max_ using performance data obtained through the test shows different levels of accuracy among the tests. Being the most similar to laboratory incremental exercise tests, the UMTT shows the largest correlation with VO_2max_ (r = 0.96) and the lowest standard error of estimate (SEE) of 2.8 mL/kg/min [10] and therefore seems the best option for estimation of VO_2max_. As originally developed for the assessment of VO_2max_ in limited spaces such as gyms, the 20mSRT also has a high level of criterion-related validity with SEE of 3.5 mL/kg/min in adults [38] and 4.7 and 5.9 mL/kg/min in healthy adults and children, respectively [39]. This indicates a higher validity for adults than for children [40]. On the other hand, both Yo-YoIRTs and 30-15IFT present lower correlation coefficients and limits of agreement with VO_2max_ and, therefore, may not be ideal for estimation of VO_2max_ [8,13,41,42]. However, it seems that criterion-related validity of the tests might be fitness level dependent as higher correlations and lower SEE of Yo-YoIRT1 were reported for recreational [43] and untrained individuals [44].

## 3. Assessment of Specific Intermittent Endurance

Soccer and most other team sports are intermittent activities with high aerobic demands [20] placed on players due to frequent changes in types of movement [31] and repetition of high-intensity running and sprinting [45]. Even though high-intensity running can be maintained throughout the match [22], the decrease in occurrences of repeated sprint sequences and number of sprints within a sequence [46] suggests the accumulation of fatigue over the course of a match which may negatively impact players’ overall physical and technical match performance [47]. As high-intensity running appears to be an important index of match-related physical performance [27], assessing player’s ability to repeat such activities and to recover from them quickly seems important. This has led to the development of the Yo-YoIRTs devised with the main purpose of assessing soccer-specific intermittent endurance [8]. Indeed, significant correlations between Yo-YoIRT1 and high-intensity running during the match have been found in young [23,48,49,50] and senior level [24,26] soccer players. Large and very large significant correlations between Yo-YoIRT1 and high-intensity running (r ranging from 0.56 to 0.76) [8,23,24,26,48,49,50], very high-intensity running (r = 0.59) [50], sprinting (r = 0.63 and 0.76) [23,49], total distance covered (r ranging from 0.53 to 0.65) [24,26,48,50] and high-intensity activity (r ranging from 0.56 to 0.77) [23,26,48,49] performed during the match seem to support the ecological validity of the test [44,51]. The same applies for the Yo-YoIRT2 as a very large correlation (r = 0.72) was obtained between Yo-YoIRT2 and peak high-intensity running in a 5-min period during the match [8]. However, the significance of these correlations has lately been brought to question [52,53,54] as these analyses were performed on pooled data from all the players in a team. This resulted in neglecting the sometimes-substantial differences in physical fitness [21,55] as well as the often-substantial differences in match running performances [21,22,56] between players from different playing positions. Namely, even though significant correlations were found on pooled data, when analyzed according to playing position, the associations between aerobic fitness and match running performances were actually trivial and non-significant, with the only exception of strikers [21]. This suggests that tactical roles dictated by playing positions as well as other contextual factors such as score line, team formation and opponent quality rather than physical fitness are primarily important in determining player’s match running performance [21,53]. Additionally, it is also interesting to notice that other field tests, such as UMTT and 20mSRT, which are not initially designed to assess specific intermittent aerobic endurance, also show large to very large correlations with high-intensity running [27,49], very high-intensity running [21,27] and high-intensity activity [49]. In fact, in young soccer players, significant correlations with high-intensity running (r = 0.70 vs. 0.65) and high-intensity activity (r = 0.75 vs. 0.73) were greater for 20mSRT than Yo-YoIRT1, raising doubt to the superiority of Yo-YoIRT1 in terms of ecological validity [49]. Generally, these findings point out that aerobic fitness is not a major limiting factor of match running performance [21,52] and that assessing specific intermittent endurance obviously does not provide an additional benefit in assessing player’s physical fitness [3].

However, when choosing the test for the purpose of aerobic fitness assessment of soccer players, the choice of the test used should be based on the player’s age, their aerobic fitness level and testing time-point. Namely, it has been shown that high-levels of VO_2_ are reached early into the Yo-YoIRT1 and that almost half of the test duration is executed with VO_2_ above 95% VO_2max_ [37]. This is quite different from the VO_2_ response elicited during continuous tests in which VO_2_ kinetics appears to be fairly linear. This means that YoYoIRTs are more metabolically demanding [8,38]. It seems that the difference between findings obtained in the continuous and intermittent tests increases as players improve their VO_2max_, their anaerobic capacity and the ability to recover quickly following a high-intensity run so that their anaerobic capacity could be expended slowly during the execution of the test. Indeed, the anaerobic contribution to the intermittent test is higher than during a continuous test as blood lactate concentrations and end-test velocities are significantly higher after the intermittent in comparison to the continuous test [17]. This possible differentiation between the 20mSRT and Yo-YoIRT1 as players get fitter is further supported by the almost perfect correlation (r = 0.89) in very young soccer players [49], while slightly lower correlations were observed for adults and elite athletes [43]. Therefore, the literature suggests that 20mSRT should be used with younger and less aerobically fit players as the protocol involves lower starting speeds and smaller increments at the beginning of the test, while Yo-YoIRT1 is more useful for aerobically fitter players and during the in-season period when certain level of conditioning has already been reached [49]. The shorter testing time for Yo-YoIRT1 in young soccer players also makes it a better option for in-season period when time devoted to testing is limited. On the other hand, a very large significant correlation obtained between 30-15IFT and mean sprint time of the RSA test (r = 0.88) [57] and Yo-YoIRT1 (r = 0.75) [58] suggest that even 30-15IFT can be used for evaluation of specific intermittent endurance even though the test was not created for that particular reason [13,42].

## 4. Performance Monitoring and Assessment of the Training Effects

Very important features of any test are its ability to detect the smallest increase in performance that might be practically significant [59] and its sensitivity to detect training effects after a training program [2,60]. In order for the test to be highly sensitive to changes over time its smallest worthwhile change (SWC) should be greater than the typical error of measurement (TE) [61]. This ensures that the change in the variable really reflects fitness improvement rather than just a variation within the subjects tested. However, the TE alone is not the best indicator of the test sensitivity to training effects, but it is the magnitude (noise) in relation to the usually observed changes (signal) in that test that matters the most [61]. The greater the signal-to-noise ratio, the likely greater sensitivity of the test to detect changes in fitness [61,62].

The TEs are generally lower in 30-15IFT [63,64,65] and Vam Eval test [66], a modified version of the UMTT, than in 20mSRT [49,67,68] and both Yo-YoIRTs [26,43,48,49,68,69,70,71,72,73,74,75,76,77] (Table 1, Table 2, Table 3, Table 4 and Table 5). This is largely due to the fact that TE is dependent on the measurement unit [78] making the speed-related tests less variable than the distance-related ones and therefore rendering a direct comparison between tests inappropriate. The Vam Eval test [66,79] and the 30-15IFT [13,42] both have 0.5 km/h increments presenting much bigger stage steps in comparison to the 40-m shuttle increments which is a minimal detectable change in all distance-related tests. This bigger increment in single stage probably also contributes to a lower variability in test-retest measures. Additionally, the uneven time-dynamic of the speed increments throughout both Yo-YoIRTs and longer time exercised at a single speed stage, requiring maintenance of a very high physiological stress for a longer time which is influenced by motivation, very likely contributes to their higher TEs in comparison to other tests. Indeed, the TEs expressed as CV for Yo-YoIRT1, Yo-YoIRT2 and 20mSRT range between 3.5% and 17.3%, 4.2% and 12.7%, and 2.2% and 6.8%, respectively, while lower values of 3.5% and from 1.5% to 2.5% were found for Vam Eval and 30-15IFT, respectively. Although the number of studies reporting TEs of UMTT and 30-15IFT in soccer players is much lower than the ones reporting TEs for the 20mSRT and Yo-YoIRTs it does appear that the TEs of speed-related tests are more stable than those of distance-related tests. It also can be noticed that the TEs expressed as CVs. within the tests are generally lower in older and fitter players. For example, lower CVs. in older groups of soccer players were reported in most studies in which direct comparisons between age groups were made [70,71], while between-study analysis reveal that recreational [43] and sub-elite [69,70] players generally present higher CVs. compared to their elite counterparts [48,49]. Accordingly, lower TE of 0.23 km/h (CV = 1.3%) for UMTT was reported in moderately trained distance runners [79] in comparison to the TE of 0.57 km/h (CV of 3.5%) found in young soccer players [66] suggesting that greater experience with the mode of running in a test can also contribute to the lower trial-to-trial variability. This is important to acknowledge as both the ability to detect SWC and the sensitivity of the test is influenced by the TE. Therefore, if reliability of the test cannot be directly assessed in a particular group of players, the practitioners are advised to use the TEs from the literature which is derived from the subjects that most closely resemble their athletes.

The “signal” or the usually observed change following a training program in soccer players is also generally greater in the distance-related tests than in the speed-related tests. Namely, the mean change following training programs comprised of different HIIT formats [18] (i.e., long interval HIIT, short interval HIIT, repeated sprint training (RST), sprint interval training (SIT) and small-sided games (SSG)) lasting from 2 to 12 weeks in soccer players averages 2.7% for UMTT or Vam Eval test [80,81,82,83,84,85] (Table 1), 6.7% for 20mSRT [67,68,86,87,88,89] (Table 2), 18.8% for Yo-YoIRT1 [58,68,74,85,90,91,92,93,94,95,96,97,98,99,100,101,102,103,104,105,106,107,108,109,110] (Table 3), 16.5% for Yo-YoIRT2 [74,77,111,112,113,114,115,116,117] (Table 4) and 9.1% for 30-15IFT [58,84,118,119,120,121] (Table 5). Although solid conclusions cannot be made due to the differences in duration and experimental designs of the studies it is interesting to notice that training programs comprised of short interval HIIT offered the greatest improvements in UMTT, 20mSRT and 30-15IFT. Namely, performing a combination of short interval HIIT and RST within a 10-week training program yielded an 8.1% or 1.3 km/h increase in UMTT end-test speed [81] while 5 weeks of short interval HIIT program resulted in 20.5% or 365 m increase in 20mSRT total distance covered [87]. Similarly, the greatest improvement in 30-15IFT was also observed following training programs comprised of short interval HIIT as 5.8% (1.3 km/h) and 28.3% (3.6 km/h) increments in end-test speeds were noticed following a 6-week training program in male amateur [84] and semi-professional female soccer players [118], respectively. The fact that short interval HIIT produced the greatest “signal” in these tests is understandable given the fact that short and long interval HIIT have shown the greatest potential to improve VO_2max_ [18,122] and 20mSRT and UMTT both have very large (r = 0.96) [10] and almost perfect (r = 0.84) [40] correlations with VO_2max_. The 30-15IFT, however, possess only large correlation with VO_2max_ (r = 0.68) [13], but its high specificity to the short interval HIIT sessions probably makes it suitable for capturing the “signal” from this type of interval training programs [42]. On the other hand, a wide range of improvements in Yo-YoIRT1 were noticed after each HIIT program. However, the greatest improvements were obtained after training programs comprised of long interval HIIT and small-sided games (SSG) [91] or a combination of both [58]. The ability of Yo-YoIRT1 to capture such “signals” is in accordance with the soccer-specific nature of the test [8]. Similarly, the greatest improvement in Yo-YoIRT2 was observed following 4 weeks of SIT [116] which is also logical considering the test’s capacity to capture larger portion of anaerobic capacity in comparison to Yo-YoIRT1 [8]. Bearing in mind that above presented conclusions could simply be a result of differences in the experimental training interventions we believe that this information could be valuable to coaches when selecting tests for the training program evaluation purposes as tests appear to differentiate between “signals” produced by different training programs. So, it seems as a good practice to select tests based on the training program chosen for evaluation. For example, significant improvement of 17.1% in Yo-YoIRT1 was obtained following 7 weeks of SSG training program even though non-significant decrements of −0.7% in VO_2max_ and 20mSRT were noticed [68]. The SSG training program obviously produced some valuable improvements important for already aerobically well-prepared soccer players which would not be captured if only 20mSRT had been employed [68].

Even though the overview of the studies conducted on soccer players indicate that field tests may differ in their ability to “receive the signal” emitted from different training programs, it is the signal-to-noise ratio that really defines the sensitivity of the test [59,60]. The most accurate measure of sensitivity is the one calculated with the TE and the change in the measure following a training program assessed within the same subject sample, i.e., within the same study. Unfortunately, there are only few intervention studies in which reliability of the tests was assessed prior to the commencement of the training program [67,68,74]. Therefore, for the most studies reported in Table 1, Table 2, Table 3, Table 4 and Table 5, sensitivity was calculated using the TE from other study done on participants with the most similar characteristics. Additionally, the number of studies available for calculation of the signal-to-noise ratio as well as the type and duration of the training programs analyzed differ significantly among the tests and, therefore, direct between-test comparison should be made with caution. Average signal-to-noise ratios were 1, 2.9, 2.7, 2.5 and 5.1 for UMTT or Vam Eval test, 20mSRT, Yo-YoIRT1, Yo-YoIRT2 and 30-15IFT, respectively, suggesting that all tests can be considered sensitive to track adaptations to training. Lower sensitivity of the UMTT or Vam Eval test might partially be due to the fact that reliability measure used for calculation of the ratios was only reported in one study on young soccer players and it turned to be higher (CV = 3.5%) [66] than the one reported on older endurance trained athletes (CV = 1.3%) [79] with more experience in continuous running.

It is possible that more experienced soccer players might exhibit better reliability of this test and consequently make it more sensitive to detect long-term adaptations. Namely, it does seem that higher reliability of the 30-15IFT is the main reason for its greater sensitivity as percentage changes after training interventions captured with this test [58,84,119,120,121] are generally quite similar to the ones captured with UMTT or Vam Eval test [81,84]. This is especially evident in Dellal et al. [84] in which both tests were used for aerobic fitness assessment. However, it is very important to emphasize once again that sensitivity of the test to detect adaptations is mostly influenced by the specificity of the training type imposed on players. For example, even though TE expressed as CV was lower in 20mSRT (4.9%) vs. Yo-YoIRT1 (9%), making the denominator much lower for calculation of signal-to-noise ratio, the adaptations to both the SSG training program and the training program incorporating prolonged short-interval HIIT and RST were better captured with Yo-YoIRT1 [68]. In this case, it appears that both training programs applied to the players were of such volume and activity patterns that it was more similar or specific to the performance of the Yo-YoIRT1 making the players better conditioned to perform on that particular test. Indeed, acute physiological responses to prolonged short-interval HIIT and extensive RST sessions are much more similar to the physiological demand of Yo-YoIRT1 in which half of the test duration is performed with the intensity of ~95% VO_2peak_ [37], therefore creating a better “signal” for that test. Similarly, 11 weeks of long-interval HIIT, SSG, sprint training and technical and tactical drills was better captured with Yo-YoIRT1 than Yo-YoIRT2 in young soccer players, again, mostly due to the large differences in the “signal” [74]. It seems that the overall training program was more focused on the improvement of aerobic capacities and less on anaerobic capacities creating the difference in the adaptation which resulted in different percentage increases in these two tests used. On the other hand, long-interval HIIT, SSG and resistance training intervention lasting for 7 weeks was similarly detected by Yo-YoIRT1 and 30-15IFT [58]. While both tests are intermittent in nature and quite similar in terms of specificity, they presented identical sensitivity probably because the overall training program was comprised of activities that evenly attacked the capacities evaluated by both tests. However, even though the number of studies reporting sensitivity presented in Table 3 and Table 5 is significantly different between Yo-YoIRT1 and 30-15IFT, and while direct comparisons should not be made due to the differences in the training programs evaluated, it does seem that the 30-15IFT presents superior overall sensitivity.

Another very important characteristic of the test is its usefulness or the ability to detect the SWC in a measure. Ideally, the TE should be less than half of the SWC and in that case any change in the test greater than the SWC would almost certainly be meaningful [60]. However, the test is rated as *good* whenever the SWC is greater than the TE and *Ok* or *medium* when the TE is equal to the SWC [123]. The TE larger than the SWC makes the test *marginal,* but even with *marginal* test we are still able to detect moderate, large and very large changes in a measure [60,123]. Namely, the changes of 1×, 3×, 6× and 10 × SWC can be considered as small, moderate, large and very large [59]. Determining the magnitude of the SWC is very complex and depends on many factors such as training context, type of adaptations that are being evaluated and the variable itself [60]. For performance variables in team sports the SWC is most often determined as 0.2× between-athletes standard deviation [59,60]. However, using between-athletes SD for calculation makes the SWC susceptible to influence by group homogeneity, i.e., more heterogeneous groups will exhibit larger SWC and may present the test as more useful. Usually, younger and less fit groups of players show more heterogeneity although this is not the general rule as different levels of heterogeneity was found between experimental groups of the same subject sample [95]. On the other hand, great homogeneity was also found in players with significantly different initial fitness status [103]. Anyway, it does appear that all field tests reviewed here present with *marginal* usefulness while the tests were rated as *Ok* and *good* mostly in studies with younger [48,49,68,71,72,73,88,89] and less fit [43,87,95] or less experienced [71,105] players with a few exceptions noticeable for Yo-YoIRT2 [75,111,115] and 30-15IFT [65]. It is advisable, therefore, that strength and conditioning coaches compare the initial fitness scores of their players with the ones presented in Table 1, Table 2, Table 3, Table 4 and Table 5 and, by choosing the most appropriate TE, estimate the potential usefulness of a particular test for their players. It is also worth noting that the reported SWCs in UMTT or Vam Eval test, Yo-YoIRT2 and 30-15IFT are most often smaller than the test’s stage increment making the improvement of only one stage in the test performance already substantial and worthwhile. Indeed, both the UMTT/Vam Eval test and 30-15IFT use 0.5 km/h increments while minimal detectable increment in Yo-YoIRT2 is 40 m making the usually observed SWCs of 0.1–0.2 km/h, 0.2–0.3 km/h and 10–45 m, respectively, almost exclusively outperformed by improvement of just one stage. On the other hand, the usually reported SWCs of 30–70 m and 40 to 135 m for 20mSRT and Yo-YoIRT1, respectively, are much larger than their stage increments of 20 and 40 m rendering one stage increment in the test performance often insufficient for practical significance.

Although VO_2max_ is not related to soccer match performance and should not be a variable of particular interest for strength and conditioning coaches they are still very often interested to find out if the VO_2max_ has increased after a training period. However, if one is still really interested in assessing VO_2max_ and its improvement following a training program, the most logical option would be to use the field test that has the greatest criterion-related validity. The UMTT or the Vam Eval test offer the greatest correlation (r = 0.96) between end-test speed and VO_2max_ and the lowest SEE of 2.81 mL/kg/min amongst the proposed field tests thus appearing as the best candidate for the job [10]. On the other hand, the 20mSRT, Yo-YoIRT1, Yo-YoIRT2 and 30-15IFT all have lower criterion-related validity with correlation coefficients of 0.84 [40], 0.74 [124], 0.47 [124] and 0.68 [13], respectively, which points out their very low capacity to accurately estimate VO_2max_, especially in top-level athletes. As usually observed changes in VO_2max_ following several weeks of HIIT in soccer players are in range of 5 to 11% or 3 to 6 mL/kg/min [20,122,125,126], even the UMTT or the Vam Eval test with their highest criterion-related validity among the field tests and high reliability (TE of 1.92 mL/kg/min) [10] are not accurate and sensitive enough to capture such small changes induced by a training intervention. Namely, adding the SEE of 2.81 mL/kg/min to the TE of 1.92 mL/kg/min, which is actually the third of the upper range value of the VO_2max_ improvement or the “signal”, increases the overall “noise” of the test and renders the test invalid to provide reliable data. Therefore, it is advised that field tests are not used for calculation of VO_2max_ and especially for evaluation of the training effects through the lens of VO_2max_ improvements.

## 5. Training Prescription

Training prescription is probably the most vital part of strength and conditioning as it involves manipulation of numerous acute training variables in order to reach the desired physiological response [18,127]. For aerobic exercise in particular, prescribing exercise intensity is the key issue, and it becomes especially challenging when prescribing long and short format HIIT [128,129]. These training formats are recognized as optimal for accumulating the most time in the zone >90%VO_2max_ per session and their acute physiological reactions are believed to be the most important for improvement of VO_2max_ [18]. Long format HIIT includes high-intensity intervals lasting from 2 to 6 min performed at 90–105% vVO_2max_, while short format HIIT includes 10 to 60-s intervals performed at 100–120% vVO_2max_ [18,127]. As this work in the zone > 90%VO_2max_ is performed in the severe intensity domain, usually above the respiratory compensation point, prescribing exercise intensity through percentage of heart rate or VO_2max_, as is often done for low and moderate-intensity aerobic exercises, is not possible. During long format HIIT the time lag of heart rate response is sometimes as long as the bout itself, so relying on heart rate to control intensity would result in performing very inefficient sessions. The problem with heart rate is even more critical during the short format HIIT as several intervals are needed to reach the desired heart rate zone. This is why vVO_2max_, or the lowest speed required to elicit VO_2max_, has emerged as the preferred method for prescribing HIIT [18]. Prescribing exercise intensity using speed for which the cardiovascular response is determined through incremental exercise testing enables better control and more precision in execution of the session.

However, because of the different locomotor nature of the protocol, VO_2max_ is attained at different velocities in each of the discussed field tests. Congruently, end-test velocities represent different physiological qualities, i.e., they are composed of different ratios of aerobic and anaerobic capacities and different degrees of neuromuscular strain which is influenced by the specific nature of the task. Therefore, only those field tests that closely mimic the locomotor activity of the specific HIIT session pose the ability to be used for training prescription. The end-test velocities can hardly be used interchangeably to prescribe HIIT sessions.

The vVO_2max_ required for continuous running is usually obtained through incremental exercise test and, although it differs slightly from the end-test velocity, those two measures are highly correlated [10]. Therefore, this end-test velocity can be used to prescribe long format HIIT because the mode of testing is very similar to the mode of the training session [18]. Namely, the incremental exercise test is performed continuously and in straight-line so no other physiological capacity except for cardiorespiratory fitness and the energetic cost of such mode of running contribute to the task [9]. Performance of the work intervals in long format HIIT sessions rely on the same capacities, so the physiological response during the training sessions will be similar as during testing. As UMTT and Vam Eval test are continuous straight-line field tests their end-test velocities are suitable to prescribe long format HIIT sessions. These tests can also be used to prescribe short format HIIT if the session is performed on the treadmill where by jumping on and off the treadmill accelerations and decelerations can easily be omitted. However, most short format HITT sessions are performed indoors with limited space available requiring introduction of numerous changes-of-directions (COD) and corresponding accelerations and decelerations. These additional actions augment the physiological response of such mode of running [14,16] and in order to accurately individualize such sessions they need to be prescribed based on the test which closely mimics such locomotor activities. Namely, using vVO_2max_ assessed through UMTT to prescribe short interval HIIT sessions performed indoors on a court usually results in very different physiological responses between players [13,42]. This is due to their differences in anaerobic capacities and neuromuscular qualities which are required to change direction and to accelerate and decelerate throughout the session [42]. This has led to the development of 30-15IFT, an intermittent shuttle test, in which the final test speed incorporates aerobic and anaerobic capacities, COD ability and the inter-effort recovery ability in the amount which is required for performance of the short format HIIT [13]. That way all athletes elicit similar physiological reactions while performing short format HIIT at identical relative intensity [13,42]. Namely, the test is highly specific to the training sessions usually performed in intermittent sports, but not to the sports [13,42], which is why it is ideal for training prescription of such sessions.

On the other hand, 20mSRT and Yo-YoIRTs are not specific to either of the HIIT formats and can hardly be used for training prescription. The 20mSRT is a continuous shuttle incremental test, so its final speed does not incorporate inter-effort recoveries making it unsuitable for prescribing short format HIIT. Namely, the variability of the cardiorespiratory response to 10-min intermittent runs was much higher when training prescription was based on the 20mSRT (10.6%) in comparison to the 30-15IFT (2.9%) rendering some subjects unable to finish the session and others below the desired heart rate zone [13]. Similarly, greater anaerobic contribution [16] and poorer running economy [14] during relative shuttle compared to the straight-line running limits the potential of the 20mSRT to be used for prescription of long format HIIT. Namely, the difference between end-test speeds in 20mSRT and UMTT can inform the coach about the COD ability of their athletes, with smaller the difference the better the COD ability [42]. However, as this difference between end-test speeds can be highly variable [130], the cardiorespiratory responses of straight-line running prescribed through the results obtained with the 20mSRT could also appear highly variable [15]. Therefore, it would be very hard to capture the ideal acute cardiorespiratory response during long format HIIT if the 20mSRT is used for training prescription.

As indicated earlier the Yo-YoIRTs are soccer-specific tests with the main purpose of evaluating player’s ability to perform intense exercise [8]. Although the tests are very similar to the short format HIIT [131], the protocol design limits their potential to be used for training prescription purposes. At the beginning of the test the speed increments are rather steep and vary in volume while the latter stages have smaller increments which are distance-regulated. Namely, for each speed stage latter in the test the athlete is required to cover the 320-m distance which subsequently shortens time spent at each stage. Additionally, each of the eight shuttles are interspersed with 10-s recovery, making it hard to assess how an athlete would actually cope with the requirement of maintaining the corresponding speed for longer as is necessary during HIIT sessions. Making the test distance-focused is contrary to the concept of HIIT prescription as training sessions are usually time-defined [18]. Therefore, when it comes to training prescription the real question which needs to be answered through testing is whether an athlete can withstand a certain speed level for the duration of an average high-intensity interval. Being a time-defined test the 30-15IFT is, therefore, the best choice to prescribe short format HIIT [13,42].

## 6. Conclusions

At the beginning aerobic field tests were developed as a cheaper and easier means for the assessment of athletes’ fitness compared to laboratory tests. However, different needs have emerged through time and have guided their development. These main goals shaped and determinate the test’s main purposes and characteristics. As safer and pacing-free alternative to the 12-min run or the Cooper test, the UMTT was born in 1980 with the main purpose of measuring VO_2max_. The necessity to assess VO_2max_ indoors with limited space available led to the development of 20mSRT which appeared to be valid and reliable alternative to the UMTT. As strength and conditioning coaches working in soccer were interested in evaluating sport-specific intermittent aerobic ability their requirement resulted in the appearance of the Yo-YoIRTs in the early 1990s. Finally, the inability of all these tests to prescribe short format HIIT, often performed with numerous COD as organized indoors, laid the ground for the birth of 30-15IFT in 2008. All these field tests have their strengths and weaknesses and should be used accordingly, i.e., a test should be selected when it fits best for the particular purpose of the testing (Table 6).

Namely, when it comes to VO_2max_ assessment, the UMTT and Vam Eval test appeared as the best solution even though it must be pointed out that VO_2max_ is not related to soccer match performance and its assessment should not be a priority in soccer players. Additionally, tracking VO_2max_ improvement through time using field tests is not very feasible due to the small magnitude of potential VO_2max_ improvements and an inadequate reliability and criterion-related validity of the tests to give them the necessary sensitivity. The findings presented herein suggest that Yo-YoIRTs are the most often used tests in soccer players. However, the findings obtained with these two, or any other field test for that matter, should not be used to predict on-field match performance as this practice seems to be misleading. The comparison of the signal-to-noise ratios suggested that 30-15IFT is the most sensitive test to track adaptations to training programs. However, this conclusion should be taken with caution as the number of studies reviewed and their methodology differ significantly between the tests reported. Anyway, strength and conditioning coaches are advised to choose the test based on the training program they are about to implement as it appears that the tests differ in their capacities to detect “signals” emitted. While all field tests present with *marginal* usefulness, the usually reported SWCs for UMTT/Vam Eval test, Yo-YoIRT2 and 30-15IFT were smaller than their stage increment making the improvement of only one stage in the test performance already worthwhile. Finally, when it comes to training prescription, UMTT and 30-15IFT should be preferably used for programing long and short HIIT, respectively.

## Figures and Tables

**Table 1:** Metric characteristics of the University of Montreal Track Test or Vam Eval test extracted from studies conducted on soccer players.

Study	Age and Gender of the Participants	Level of the Participants	Typical Error of Measurement Expressed as Coefficient of Variation	Typical Error of Measurement (Noise)	Smallest Worthwhile Change (0.2× between Subjects SD)	Usefulness of the Test	Training Type and Duration	Initial Level	Usually Observed Change (Signal) Following a Training Program	Usually Observed Change (Signal) Following a Training Program	Signal-to-Noise Ratio
Buchheit et al. (2013) [66] ^b^	14.5 ± 1.5 M	Elite	3.5%	0.57 km/h	0.22 km/h	Marginal		≈16.2 km/h			
Los Arcos et al. (2015) [80] ^a^	15.5 ± 0.6 M	National, elite			0.18 km/h	Marginal	HIIT l (6 w)	16.8 km/h	1.7%	0.3 km/h	0.5
Dupont et al. (2004) [81] ^a^	20.2 ± 0.7 M	National, elite, professional			0.16 km/h	Marginal	HIIT s + RST(10 w)	16.1 km/h	8.1%	1.3 km/h	2.3
Faude et al. (2014) [82] ^c^	16.5 ± 0.8 M	High-level, professional conditions			0.2 km/h	Marginal	HIIT s (4 w)	17.8 km/h	−2.8%	−0.5 km/h	−0.8
Faude et al. (2013) [83] ^c^	15.9 ± 0.8 M	High-level, professional conditions			0.21 km/h	Marginal	HIIT s (5.5 w)	17.1 km/h	1.5%	0.25 km/h	0.4
Dellal et al. (2012) [84] ^b^	26.3 ± 4.7 M	Amateur			n/a	/	HIIT s (6 w)	≈15.8 km/h	6.6%	≈1 km/h	1.9
Wong et al. (2010) [85] ^b^	24.6 ± 1.5 M	Elite, professional			0.04 km/h	Marginal	HIIT s (8 w)	15.9 km/h	3.1%	0.5 km/h	0.9
Faude et al. (2014) [82] ^c^	15.9 ± 0.8 M	High-level, professional conditions			0.2 km/h	Marginal	SSG (4 w)	17.5 km/h	1.7%	0.3 km/h	0.5
Los Arcos et al. (2015) [80] ^a^	15.5 ± 0.6 M	National, elite			0.16 km/h	Marginal	SSG (6 w)	17.0 km/h	−0.6%	−0.1 km/h	−0.2
Dellal et al. (2012) [84] ^b^	26.3 ± 4.7 M	Amateur			n/a	/	SSG (6 w)	≈16.1 km/h	5.1%	≈0.8 km/h	1.5

Legend: ^a^: University of Montreal Track Test was used in the study, ^b^: Vam Eval test was used in the study, ^c^: the type of maximal incremental exercise test used in the study was not clearly defined, M: male, HIIT: high-intensity interval training, HIIT s: short format HIIT, HIIT l: long format HIIT, RST: repeated sprint training, SSG: small-sided games, w: weeks. The study from which the TE was taken for calculation of the signal-to-noise ratio is indicated in the brackets.

**Table 2:** Metric characteristics of the multistage 20-m shuttle run test extracted from studies conducted on soccer players.

Study	Age and Gender of the Participants	Level of the Participants	Typical Error of Measurement Expressed as Coefficient of Variation	Typical Error of Measurement (Noise)	Smallest Worthwhile Change (0.2× between Subjects SD)	Usefulness of the Test	Training Type and Duration	Initial Level	Usually Observed Change (Signal) Following a Training Program	Usually Observed Change (Signal) Following a Training Program	Signal-to-Noise Ratio
Aziz et al. (2005) [67]	27.2 ± 3.3 M	Elite, national team	2.2%	46 m	36 m	Marginal	n/a (5 w)	2.280 m	7.9%	180 m	3.6
Castagna et al. (2010) [49]	14.4 ± 0.1 M	Elite	3.6%	59.5 m	73.4 m	Good		1653 m			
Slettaløkken and Rønnestad (2014) [86]	18–26 M	Semi-professional			49.6 m		HIIT l (6 w)	≈2.433 m	−6.4%	−155 m	−2.9 [67]
Hill-Hass et al. (2009) [68]	14.6 ± 0.9 M	Elite	4.9%	40 m	26.2 m	Marginal	HIIT s + RST (7 w)	2.258 m	3.1%	69 m	0.6
Sanchez-Sanchez et al. (2019) [87]	22.5 ± 2.2 M	Amateur			86.2 m	Good	HIIT s (5 w)	1.770 m	20.5%	362 m	5.7 [49]
Tønnessen et al. (2011) [88]	16.4 ± 0.9 M	Elite			57.6 m	Ok	RST (10 w)	2.360 m	5.7%	144 m	2.6 [67]
Shalfawi et al. (2013) [89]	19.4 ± 4.4 F	Elite			61.6 m	Ok	RST + AgT (10 w)	1.780 m	16.8 %	264 m	4.7 [49]
Hill-Hass et al. (2009) [68]	14.6 ± 0.9 M	Elite	4.9%	40 m	48 m	Ok	SSG (7 w)	2.222 m	−0.7%	−16 m	−0.1

Legend: F: female, M: male, HIIT: high-intensity interval training, HIIT s: short format HIIT, HIIT l: long format HIIT, RST: repeated sprint training, SSG: small-sided games, w: weeks, AgT: agility training. The study from which the TE was taken for calculation of the signal-to-noise ratio is indicated in the brackets.

**Table 3:** Metric characteristics of the Yo-Yo intermittent recovery test level 1 extracted from studies conducted on soccer players.

Study	Age and Gender of the Participants	Level of the Participants	Typical Error of Measurement Expressed as Coefficient of Variation	Typical Error of Measurement (Noise)	Smallest Worthwhile Change (0.2× between Subjects SD)	Usefulness of the Test	Training Type and Duration	Initial Level	Usually Observed Change (Signal) Following a Training Program	Usually Observed Change (Signal) Following a Training Program	Signal-to-Noise Ratio
Deprez et al. (2014) [69]	12.5 ± 0.6 14.0 ± 0.5 16.2 ± 0.6 M	Sub-elite and Non-elite	U13: 17.3% U15: 16.7% U17: 7.9%	U13: 154 m U15: 171 m U17: 123 m	U13: 70.8 mU15: 88.8 mU17: 95.6 m	Marginal Marginal Marginal		U13: 890 m U15: 1.022 m U17: 1.556 m			
Deprez et al. (2015) [70]	13.9 ± 0.5 16.2 ± 0.6 18.1 ± 0.4 M	High-level	U15: 6.8% U17: 4.3% U19: 4.1%	U15: 137 m U17: 101 m U19: 107 m	U15: 94 m U17: 69.4 m U19: 67.4 m	Marginal Marginal Marginal		U15: 2.024 m U17: 2.404 m U19: 2.547 m			
Castagna et al. (2019) [71]	11.1 ± 0.9 M	2 years’ experience	5.1%	51.7 m	90.4 m	Good		1.013 m			
Krustrup et al. (2003) [26]	28 M	Elite	4.9%	91.5 m	14.4 m	Marginal		1.867 m			
Póvoas et al. (2016) [72]	9.7 ± 0.7 F	Regional level competition	10.1%	71.2 m	63.2 m	Ok		705 m			
Póvoas et al. (2016) [73]	9.7 ± 0.7 M	Regional level competition	11.1%	121.9 m	134.4 m	Ok		1.098 m			
Thomas et al. (2006) [43]	24.4 ± 6.0 M	Recreational level	8.7%	107 m	97.6 m	Ok		1.030 m			
Castagna et al. (2010) [49]	14.4 ± 0.1 M	Elite	3.8%	28.9 m	56.6 m	Good		760 m			
Castagna et al. (2009) [48]	14.1 ± 0.2 M	Elite	3.5%	29.5 m	70.4 m	Good		842 m			
Impellizzeri et al. (2008) [90]	17.8 ± 0.6 M	High level			n/a	/	HIIT l (4 w)	≈1.890 m	12%	n/a	1.6 [74]
Özcan et al. (2018) [91]	18.5 ± 1.5 M	Amateur, regional level			71.9 m	Marginal	HIIT l (6 w)	1.057.7 m	89.1%	769 m	10.2 [43]
Ferrari Bravo et al. (2008) [92]	21.1 ± 5.1 M	Professional and amateur			65.8 m	Marginal	HIIT l (7 w)	1.846 m	12.5%	231 m	1.7 [74]
Fanchini et al. (2014) [74]	17 ± 1 M	Professional, 4th national division	7.3%	140 m	66.9 m	Marginal	HIIT l + RST + SSG(11 w)	1.911 m	14.5%	277 m	1.9
Buchheit and Rabbani (2014) [58]	15.4 ± 0.5 M	National level			51.4 m	Marginal	HIIT li + SSG (8 w)	1.031 m	35%	360.9 m	4.8 [74]
Arslan et al. (2020) [93]	14.2 ± 0.5 M	Regional level			15 m	Marginal	HIIT s (5 w)	1.240 m	16.4%	244 m	2.2 [74]
Wong et al. (2010) [85]	24.6 ± 1.5 M	Elite, professional			15 m	Marginal	HIIT s (8 w)	1510 m	19.7%	298 m	2.7 [74]
Ouerghi et al. (2014) [94]	22.9 ± 1.7 M	Amateur players, 3rd national division			n/a		HIIT s (12 w)	≈1.440 m	≈70%	1.6 km/h≈1024 m	8 [43]
Hill-Hass et al. (2009) [68]	14.6 ± 0.9 M	Elite	9%	116 m	51.2 m	Marginal	HIIT s + RST (7 w)	1.764 m	21.9%	387 m	2.4
Taylor et al. (2016) [95]	24.1 ± 4.1 M	Semi-professional			54.8 m	Marginal	RST Sl (2 w)	1.830 m	24%	439 m	3.3 [74]
Taylor et al. (2016) [95]	24.1 ± 4.1 M	Semi-professional			120 m	Ok	RST COD (2 w)	1.691 m	31%	524 m	4.2 [74]
Beato et al. (2019) [96]	21 ± 2.4 M	Amateur			73 m	Marginal	RST Sl (2 w)	1.642 m	11%	180 m	1.5 [74]
Beato et al. (2019) [96]	21 ± 2.4 M	Amateur			71.8 m	Marginal	RST COD (2 w)	1.686 m	7.4%	124 m	1 [74]
Soares-Caldeira et al. (2014) [97]	21.4 ± 5.5 M	Professional futsal, regional level			72.6 m	Marginal	RST (4 w)	1.280 m	31.2%	373 m	4.3 [74]
Kavaliauskas et al. (2017) [98]	22 ± 8 M	Semi-professional			81.8 m	Marginal	RST uphill 7% (6 w)	1.468 m	11.9%	175 m	1.6 [74]
Eniseler et al. (2017) [99]	16.9 ± 1.1 M	Elite, national level			50.4 m	Marginal	RST (6 w)	2.306.6 m	7.5%	173.4 m	1 [74]
Ferrari Bravo et al. (2008) [92]	21.1 ± 5.1 M	Professional and amateur			87.8 m	Marginal	RST (7 w)	1.917 m	28.1%	538 m	3.8 [74]
Nedrehagen and Saeterbakken (2015) [100]	19.9 ± 2.5 F 22.0 ± 2.7 M	Semi-professional female and amateur male			37.6 m	Marginal	RST (8 w)	1.455 m	15.3%	222 m	2.1 [74]
Shalfawi et al. (2013) [101]	21.2 ± 2.6 F	Elite			58.6 m	Marginal	RST Sl (8 w)	920 m	27.5%	253 m	3.8 [74]
Shalfawi et al. (2013) [101]	21.2 ± 2.6 F	Elite			54.8 m	Marginal	RST COD (8 w)	1.025 m	9.3%	95 m	1.3 [74]
Beato et al. (2019) [102]	18–21 M	Elite			44.6 m	Marginal	RST Sl (8 w)	2.472 m	5.3%	132 m	0.7 [74]
Beato et al. (2019) [102]	18–21 M	Elite			49.2 m	Marginal	RST COD (8 w)	2.500 m	7.8%	196 m	1.1 [74]
Sanchez-Sanchez et al. (2019) [103]	14.4 ± 0.5 M	Regional level			65.9 m	Marginal	RST COD(8 w)	914 m	8.1%	71 m	1.1 [74]
Sanchez-Sanchez et al. (2019) [103]	14.7 ± 0.5 M	Regional level			66.7 m	Marginal	RST COD (8 w)	1.764 m	2%	34 m	0.3 [74]
Campos-Vazquez et al. (2015) [104]	18.1 ± 0.8 M	Top-level national			60.4 m	Marginal	RST + ST (8 w)	2.297 m	3.5%	80 m	0.5 [74]
Haugen et al. (2014) [105]	17 ± 1 F & M	High-school level			133.8 m	Ok	RST (9 w)	1.583 m	17.4%	275 m	2.4 [74]
Nyberg et al. (2016) [106]	23.5 ± 4.0 M	Semi-professional, 2nd national league			66 m	Marginal	RST (9 w)	1.803 m	11.6%	324 m	1.6 [74]
Hostrup et al. (2019) [107]	24.9 ± 5.4 M	Sub-elite, 2nd amateur league			111.4 m	Marginal	RST (10 w)	1.910 m	1.6%	30 m	0.2 [74]
Macpherson and Weston (2015) [108]	25 ± 4 M	Semi-professional			98.6 m	Marginal	SIT (2 w)	1.523 m	18.1%	275 m	2.5 [74]
Howard & Stavrianeas (2017) [109]	15.1 ± 0.8 M	High-shool level			61.5 m	Marginal	SIT (10 w)	741.6 m	44%	326 m	6 [74]
Arslan et al. (2020) [93]	14.2 ± 0.5 M	Regional level			30.4 m	Marginal	SSG (5 w)	1.284 m	12.8%	188 m	1.8 [74]
Eniseler et al. (2017) [99]	16.9 ± 1.1 M	Elite, national level			77.6 m	Marginal	SSG (6 w)	2.320 m	4.8%	112 m	0.7 [74]
Özcan et al. (2018) [91]	18.4 ± 1.5 M	Amateur, regional level			73.6 m	Marginal	SSG (6 w)	1.235.5 m	63.1%	711 m	7.2 [43]
Hill-Hass et al. (2009) [68]	14.6 ± 0.9 M	Elite	9%	116 m	69 m	Marginal	SSG (7 w)	1.488 m	17.1%	254 m	1.9
Dello Iacono et al. (2019) [110]	18.6 ± 0.6 M	International level			27.6 m	Marginal	SSG (8 w)	1.646 m	20,9%	344 m	2.9 [74]

Legend: F: female, M: male, HIIT: high-intensity interval training, HIIT s: short format HIIT, HIIT l: long format HIIT, SIT: sprint interval training, RST: repeated sprint training, SSG: small-sided games, w: weeks, COD: change of direction, Sl: straight-line, ST: strength training. The study from which the TE was taken for calculation of the signal-to-noise ratio is indicated in the brackets.

**Table 4:** Metric characteristics of the Yo-Yo intermittent recovery test level 2 extracted from studies conducted on soccer players.

Study	Age and Gender of the Participants	Level of the Participants	Typical Error of Measurement Expressed as Coefficient of Variation	Typical Error of Measurement (Noise)	Smallest Worthwhile Change (0.2× between Subjects SD)	Usefulness of the Test	Training Type and Duration	Initial Level	Usually Observed Change (Signal) Following a Training Program	Usually Observed Change (Signal) Following a Training Program	Signal-to-Noise Ratio
Enright et al. (2018) [75]	18.3 ± 0.2 M	Elite	4.2%	34 m	31,2 m	Ok		920 m			
da Silva et al. (2011) [76]	14 ± 0.8 M	Regional level	11%	49 m	13.6 m	Marginal		445.5 m			
Thomas et al. (2006) [43]	24.4 ± 6.0 M	Recreational level	12.7%	41 m	22 m	Marginal		325 m			
Krustrup et al. (2006) [77]	22–30 17–35 M	Healthy and elite	9.6%	65.5 m	9.2 m	Marginal	Socc T (8 w)	730 m	42%	n/a	4.4
Fanchini et al. (2014) [74]	17 ± 1 M	Professional, 4th national division	7.1%	53.5 m	33.2 m	Marginal	HIIT l + RST + SSG (11 w)	718 m	8.8%	71 m	1.2
Iaia et al. (2017) [111]	17.0 ± 1.0 M	Sub-elite			33.8 m	Ok	RST sh. rest (5 w)	1.000 m	11.4%	111 m	2.7 [75]
Iaia et al. (2017) [111]	17.0 ± 1.0 M	Sub-elite			43.4 m	Good	RST lo. rest (5 w)	1.016 m	6.5%	56 m	1.5 [75]
Sagelv et al. (2019) [112]	16–19 M	High-level national			n/a	/	RST (22 w)	≈890 m	9.1%	/	2.2 [75]
Christensen et al. (2011) [113]	23.4 ± 3.5 M	Elite, 3rd national level			11.2 m	Marginal	SIT + SSG (2 w)	937 m	6.1%	57 m	1.5 [75]
Thomassen et al. (2010) [114]	23.4 ± 0.8 M	Elite			11.2 m	Marginal	SIT + SSG (2 w)	937 m	6.1%	57 m	1.5 [75]
Iaia et al. (2015) [115]	18.5 ± 1 M	Professional, national level			37 m	Ok	SIT (2′ rest) (3 w)	927 m	10.1%	93 m	2.4 [75]
Iaia et al. (2015) [115]	18.5 ± 1 M	Professional, national level			45.2 m	Good	SIT (40″ rest) (3 w)	989 m	3.8%	37 m	0.9 [75]
Mohr and Krustrup (2016) [116]	19 ± 1 M	Sub-elite, university level			13.6 m	Marginal	SIT (4 w)	680 m	49.7%	298 m	7 [74]
Ingebrigtsen et al. (2013) [117]	16.9 ± 0.6 M	Elite			26.6 m	Marginal	SIT (6 w)	559 m	11.3%	63 m	1.6 [74]
Mohr and Krustrup (2016) [116]	19 ± 1 M	Sub-elite, university level			10.4 m	Marginal	SSG (4 w)	693 m	25.8%	165 m	3.6 [74]

Legend: F: female, M: male, HIIT: high-intensity interval training, HIIT s: short format HIIT, HIIT l: long format HIIT, SIT: sprint interval training, RST: repeated sprint training, SSG: small-sided games, Socc T: regular soccer training, w: weeks. The study from which the TE was taken for calculation of the signal-to-noise ratio is indicated in the brackets.

**Table 5:** Metric characteristics of the 30-15 intermittent fitness test extracted from studies conducted on soccer players.

Study	Age and Gender of the Participants	Level of the Participants	Typical Error of Measurement Expressed as Coefficient of Variation	Typical Error of Measurement (Noise)	Smallest Worthwhile Change (0.2× between Subjects SD)	Usefulness of the Test	Training Type and Duration	Initial Level	Usually Observed Change (Signal) Following a Training Program	Usually Observed Change (Signal) Following a Training Program	Signal-to-Noise Ratio
Čović et al. (2016) [63]	22.8 ± 4.3 F	Elite	1.8%	0.31 km/h	0.2 km/h	Marginal		17.1 km/h			
Thomas et al. (2016) [64]	25.5 ± 4.3 M	Semi-professional	2.5%	1.0 km/h	0.7 km/h	Marginal		n/a			
Valladares-Rodríguez et al. (2017) [65]	24.4 ± 5.6 M 23.3 ± 4.5 F	Professional futsal players	M: 1.5% M F: 1.5% F	M: 0.32 km/h F: 0.21 km/h	M: 0.34 km/hF: 0.26 km/h	Ok Ok		M: 20.2 km/h F: 17.4 km/h			
Buchheit and Rabbani (2014) [58]	15.4 ± 0.5 M	National level			0.22 km/h	Marginal	HIIT l + SSG (8 w)	17.4 km/h	7%	1.2 km/h	4.7 [65]
Dellal et al. (2012) [84]	26.3 ± 4.7 M	Amateur			n/a	/	HIIT s (6 w)	≈19.4 km/h	5.8%	≈1.3 km/	3.9 [65]
Arazi et al. (2017) [118]	23.4 ± 1.3 F	Semi-professional, regional level			0.7 km/h	Marginal	HIIT s (6 w)	12.7 km/h	28.3%	3.6 km/h	11.3 [64]
Paul et al. (2019) [119]	16.2 ± 0.8 M	National level			0.22 km/h	Marginal	HIIT s + SSG (4 w)	17 km/h	8.2%	1.4 km/h	5.5 [65]
Rabbani et al. (2019) [120]	24.1 ± 3.723.2 ± 2.2 M	Semi-professional, 2nd national level			0.22 km/h 0.24 km/h	Marginal	HIIT s + SSG (4 w)	19.5 km/h19.2 km/h	6.9% & 6.2%	1.3 & 1.2 km/h	4.6 [65] 4.1 [65]
Dellal et al. (2012) [84]	26.3 ± 4.7 M	Amateur			n/a	/	SSG (6 w)	≈19.5 km/h	5.1%	≈1 km/h	3.4 [65]
Campos-Vazquez et al. (2017) [121]	27.7 ± 4.3 M	Professional, 2nd national level			0.16 km/h	Marginal	Socc T + M (4 w)	20.1 km/h	5%	1 km/h	3.3 [65]

Legend: F: female, M: male, HIIT: high-intensity interval training, HIIT s: short format HIIT, HIIT l: long format HIIT, SSG: small-sided games, Socc T: regular soccer training, M: match, w: weeks. The study from which the TE was taken for calculation of the signal-to-noise ratio is indicated in the brackets.

**Table 6:** Advantages and disadvantages of the reviewed field aerobic fitness tests.

Field Aerobic Fitness Test	Advantages	Disadvantages
UMTT/Vam Eval	Moderate to high reliability High criterion-related validity—best solution for the assessment of VO_2max_ SWC smaller than one stage of the test Best for prescription of long format HIIT	Low to moderate sensitivity Marginal usefulness Athletic track required for testing
20mSRT	Short-distance course required for testing Low end-test running speeds Short testing time High sensitivity Ok to good usefulness	Low to moderate reliability Moderate criterion-related validity for the assessment of VO_2max_ SWC larger than one stage of the test Unsuitable for training prescription
Yo-YoIRT1	Short-distance course required for testing High sensitivity	Low reliability Low criterion-related validity for the assessment of VO_2max_ Marginal usefulness SWC larger than one stage of the testUnsuitable for training prescription
Yo-YoIRT2	Short-distance course required for testing High sensitivity Very short testing time Medium usefulness SWC smaller than one stage of the test Appropriate for players with high aerobic and anaerobic fitness	Low reliability Very low criterion-related validity for the assessment of VO_2max_ Not appropriate for players with low aerobic fitness Unsuitable for training prescription
30-15IFT	Medium-size-distance course required for testing High reliability Excellent sensitivity Medium usefulness SWC smaller than one test stage Best for prescription of short format HIIT	Low criterion-related validity for the assessment of VO_2max_

Legend: UMTT: University of Montreal Track test, 20mSRT: 20-metre shuttle run test, YoYoIRT1: YoYo intermittent recovery test level 1, YoYoIRT2: YoYo intermittent recovery test level 2, 30-15IFT: 30-15 intermittent fitness test, SWC: smallest worthwhile change, HIIT: high-intensity training.
